# Supplementary material for: Highway to Cell: Selection of the Best Cell-Penetrating Peptide to Internalize the CFTR-Stabilizing iCAL36 Peptide
Source: Pharmaceutics. 2022 Apr 7;14(4):808. doi: 10.3390/pharmaceutics14040808 (PMC9032934; doi:10.3390/pharmaceutics14040808)
Supplement: Supplementary file 1 [file pharmaceutics-14-00808-s001.zip › pharmaceutics-1622119-supplementary materials.pdf]

pharmaceutics

Supplementary Materials: Highway to Cell: Selection of the Best Cell-Penetrating Peptide to Internalize the CFTR Stabilizing iCAL36 Peptide

Seisel Quentin, Lakumpa Israpong, Josse Emilie, Vivès Eric[2], Varilh Jessica, Taulan-Cadars Magali and Boisguérin Prisca

1. Materials and Methods

1.1. Circular Dichroism (CD) Measurements.

CD spectra were recorded on a Jasco 810 (Japan) dichrograph in quartz suprasil cells (Hellma) with an optical path of 1 mm for the peptides alone or in the presence of liposome vesicles. The same concentration of peptide (65 µM) was used for each condition. Spectra were obtained from 3 accumulations between 190 and 260 nm with a data pitch of 0.5 nm, a bandwidth of 1 nm, and a standard sensitivity. Raw data were corrected by subtracting the blank signal (milliQ water, liposomes alone, 50% TFE) and then converted to molar ellipticity per residue.

Measurements in the presence of LUV. The spectrum of a 65 µM solution of CPP-iCAL36 in milliQ water was first measured in the absence of large unilamellar vesicles (LUV). LUV preparation is described below. Then LUVs were added to obtain LUV/peptide ratios of 2:1 and 10:1. The conversion to molar ellipticity per residue was made considering the dilution caused by the addition of LUV.

Measurements in the presence of TFE. 100 µL of a 130 µM CPP-iCAL36 solution in milliQ water was mixed to 100 µL 2,2,2-Trifluoroethanol (TFE, Sigma-Aldrich) (final peptide concentration: 65 µM) and the signal of the resulting mixture was measured.

1.2. Liposome Leakage Assay

Dioleylphosphatidylcholine (DOPC) phospholipids, sphingomyelin (SM), and cholesterol (Chol) were purchased from Avanti Polar Lipids. The lipids were dissolved in organic solvent (chloroform/methanol, 3/1) and mixed at a ratio of DOPC/SM/Chol (4/4/2; mol/mol/mol). Large unilamellar vesicles (LUV) were prepared by removing the organic solvent (evaporation for 45-60 min at 60°C) and rehydrating of lipids in buffer (20 mM HEPES, 75 mM NaCl, pH 7.4) containing 12.5 mM ANTS fluorescent dye (8-aminonaphthalene-1,3,6-trisulfonic acid, disodium salt; Invitrogen) together with 45 mM DPX quencher (p-xylene-bispyridinium bromide; Invitrogen). The suspension was vigorously agitated with a Vortex (30 s), freeze-thawed 5 times, and then extruded 21 times through two stacked 100 nm polycarbonate filters (Nucleopore, Whatman). Free dye and quencher were removed by gel filtration (G50-sepharose, Amersham Biosciences). LUV concentration was assessed using the LabAssay Phospholipid kit (Wako) as described by the manufacturer and LUV mean size was determined by Dynamic Light Scattering (DLS, NanoZS, Malvern).

Fluorescence leakage assay was measured on a PTI spectrofluorometer at room temperature (Ex = 360 nm ± 3 nm; Em 530 nm ± 5 nm). In detail, LUVs were diluted in 1 mL buffer (20 mM HEPES, 145 mM NaCl, pH 7.4) to a final concentration of 100 µM. To access the background fluorescence, the LUVs alone were measured for 100 seconds. Thereafter, leakage was measured as an increase in fluorescence intensity upon addition of iCAL36 or a CPP-iCAL36 conjugate (2.5 µM final concentration) during the next following 900 seconds (15 min). Finally, 100% fluorescence was achieved by solubilizing the membranes with 0.1% (v/v) Triton X-100 resulting in a completely unquenched probe (at 1,000 seconds). The relative percentage of leakage was calculated using the following equation: [(exp. value – minimal value) / (maximal triton value – minimal value)] x 100.

1.3. Fluorescence Polarization (FP) for Ki Determination

Proteins corresponding to the PDZ domains of CAL, N2P1, and N2P2 were expressed, purified, and concentrated as described previously [8] and were kindly provided by the team of Prof. D.R. Madden (Dartmouth University).

For the Ki measurement by FP, a protein solution (e.g. CALP) was incubated 30 min at room temperature in 25 mM of Tris buffer, pH 7.5 containing 0.1 mg/mL bovine IgG (Sigma-Aldrich), 0.5 mM Thesit (Fluka) (= FP buffer) and 500 nM fluorescent ligand (example: Tamra-iCAL36). An aqueous solution of unlabeled competitor peptide (example: TatRI-iCAL36) was prepared at 5 mM in the presence of 25% DMSO (Sigma-Aldrich) and then serially diluted in a milliQ water/DMSO mixture (75:25; vol/vol). 6 µL of each serial dilution was then mixed with 54 µL of fluorescent peptide/ligand mixture (final: 60 µL, 2.5% DMSO). The final protein concentration was 1.8 x K_D.

Two solutions containing 500 nM fluorescent ligand and 2.5% DMSO in FP buffer were prepared: in the second solution, enough protein was added to fully complex the fluorescent ligand (see polarization of protein/ligand complexes (P_{PL}). The diluted solutions were incubated for 15 min at room temperature, then 3x 20 µL of each solution was transferred to a black 384-well plate designed for fluorescence measurement (Falcon). Fluorescence polarization was measured (λex: 544 nm / λem: 590 nm) using a PolarStar Omega plate reader (BMG Labtech) at 24°C and then plotted against competitor peptide concentration.

The theoretical free protein concentration [P]calc and the theoretical polarization value Pcalc were calculated according to the following equations [50]:

(1) $$[P]_{calc} = -\frac{a}{3} + \frac{2}{3} * (\sqrt{a^2 - 3b}) * \cos\left(\frac{\theta}{3}\right)$$

(2) $$\theta = \arccos\left[\frac{-2a^3+9ab-27c}{2 * (a^2-3b)^{1,5}}\right]$$

(3) $$a = K_D + Ki + [L]_0 + [C]_0 - P_0$$

(4) $$b = Ki * ([L]_0 - [P]_0) + K_D * ([C]_0 - [P]_0) + K_D * Ki$$

(5) $$c = -K_D * Ki * [P]_0$$

(6) $$P_{calc} = \left(P_L + [P]_{calc} * \frac{P_{PL}}{K_D}\right) / \left(1 + \frac{[P]_{calc}}{K_D}\right)$$

$[L]_0$: initial concentration of the fluorescent ligand

$[P]_0$: initial concentration of the protein

$[C]_0$: initial concentration of the peptide competitor

K_D: dissociation constant of the proteine/fluorescent ligand interaction

Ki: inhibition constant of the proteine/peptide competitor interaction

P_L: polarization of the ligand alone ($[P]_0 = 0$)

P_{PL}: polarization of protein/ligand complex (maximal $[P]_0$)

The Ki of the protein/peptide competitor interaction was then determined by the least square method, varying the Ki in such a way as to minimize the discrepancy between the experimental and theoretical polarization values (Microsoft Excel SOLVER plugin).

2. Results

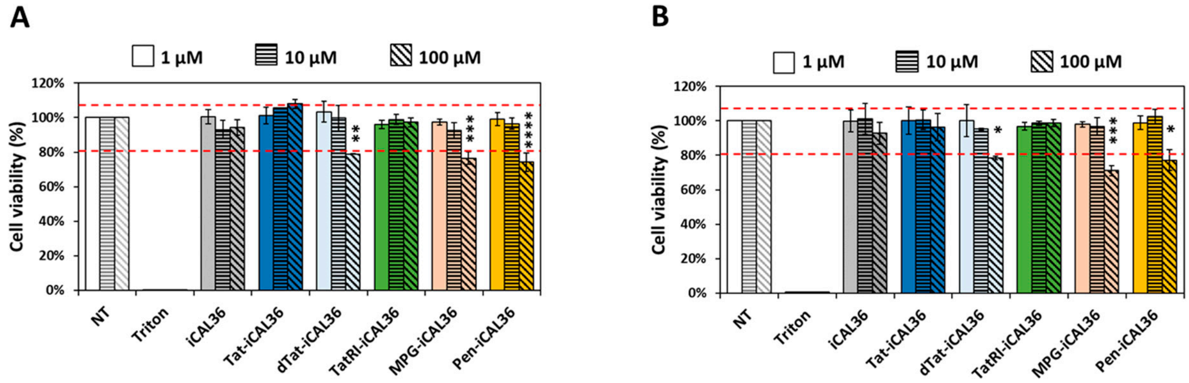

Figure S1. Cellular viability of Caco-2 (A) and Calu-3 (B) cells after CPP-iCAL36 incubation. Both cell lines were incubated with iCAL36, Tat-iCAL36, dTat-iCAL36, TatRI-iCAL36, MPG-iCAL36 and Pen-iCAL36 at the indicated concentrations. Non-transfected cells were used as negative control and Triton-treated cells were used as positive control. A statistically significant difference was noticed between the non-treated cells (NT) and 100 µM dTat-iCAL36, 100 µM MPG-iCAL36 and 100 µM Pen-iCAL36 (one-way ANOVA, Bonferroni's post-test). The viability for all other transfected conditions did not show statistically significant (ns) differences compared to non-treated cells (not shown in the figure). Graphical representation of data obtained from duplicates from three independent cell cultures (mean ± SD, n = 3). (Statistical relevance was given as *p < 0.05; **p < 0.01; ***p < 0.001; ****p < 0.0001 and ns > 0.05).

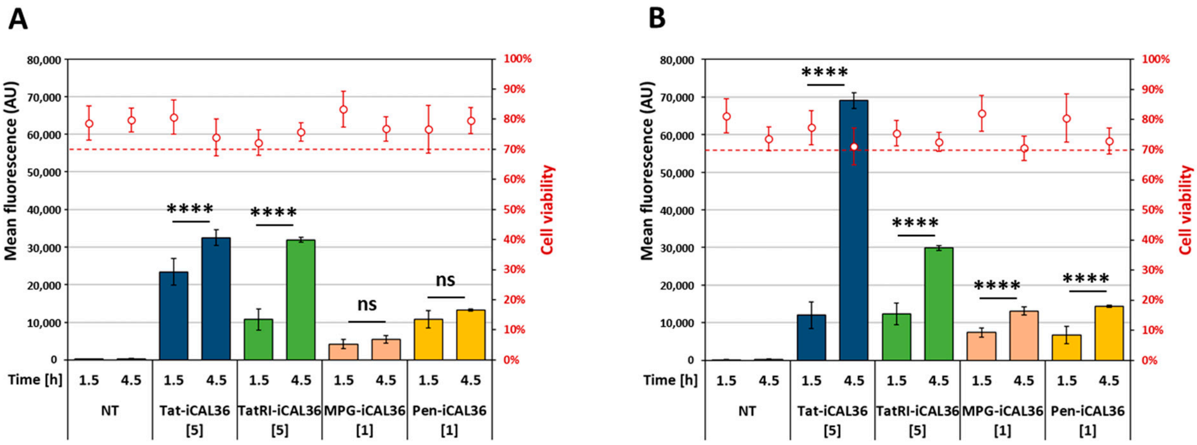

Figure S2. Time-dependent cellular internalization of CPP-iCAL36 in Caco-2 (A) and Calu-3 (B) cells. Both cell lines were incubated with iCAL36, Tat-iCAL36, dTat-iCAL36, TatRI-iCAL36, MPG-iCAL36 and Pen-iCAL36 at the concentrations indicated in the brackets [µM] for 1.5h or 4.5 h. Non-transfected cells (NT) were used as negative control. After cell trypsinization (to remove membrane externally bound peptides) fluorescence and cell viability were acquired by flow cytometry. (not shown in the figure). Graphical representation of data obtained from duplicates from three independent cell cultures (mean ± SD, n = 3). A statistically significant difference was noticed between the different incubation times for Tat-iCAL36 and TatRI-iCAL36 in Caco-2 cells as well as for all CPP-iCAL36 conjugates in Calu-3 cells (2way ANOVA, Bonferroni's post-test, statistical relevance was given as ****p < 0.0001 and ns > 0.05).

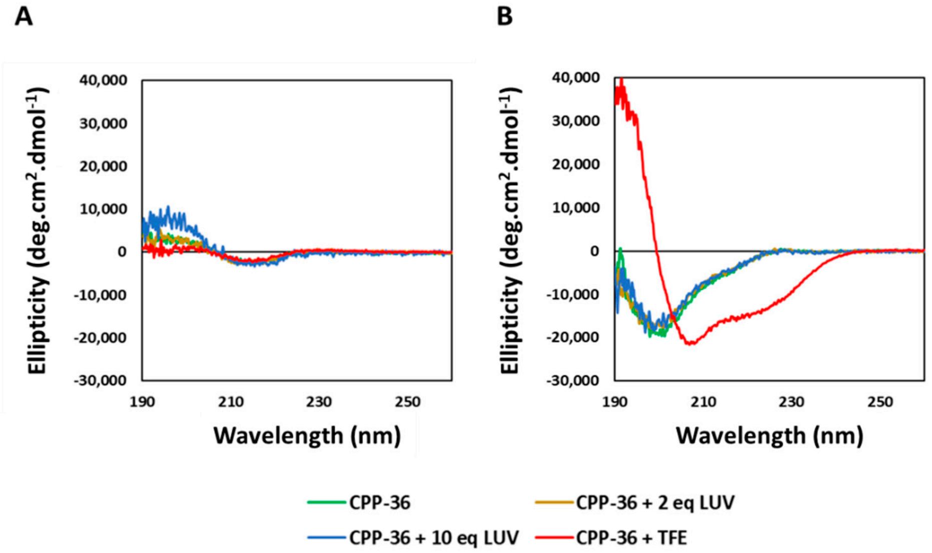

Figure S3. CD spectra of TatRI-iCAL36 (**A**) and Pen-iCAL36 (**B**). Circular dichroism (CD) profiles of TatRI-iCAL36 and Pen-iCAL36 alone (65 μM), in the presence of 2 or 10 equimolar LUVs composed of DOPC/SM/Chol (4/4/2; mol/mol/mol) or of 50% 2,2,2-Trifluoroethanol (TFE).

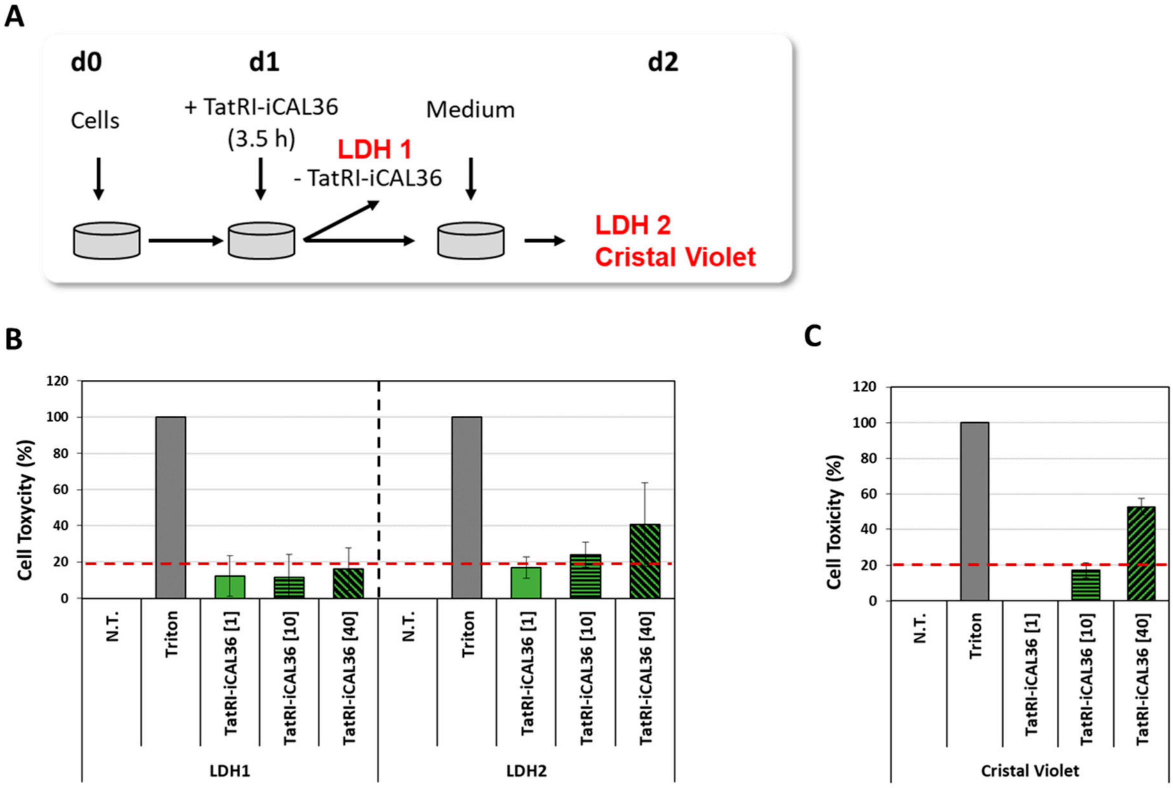

Figure S4. Evaluation of 16HBE cell toxicity (LDH1 and LDH2) and viability (Cristal Violet) after TatRI-iCAL36 incubation.

<u>(**A**) Schematic representation of the protocol:</u> 24h At day zero (d0) cells were seeded. Next day (d1), human bronchial epithelial cells 16HBEge-p.Phe508del were incubated in a dose-dependent manner with TatRI-iCAL36 (concentrations were given in brackets [μM]) for 3.5 h. First LDH assay was performed using 50 μL supernatant before TatRI-iCAL36 removing. Fresh medium was added to the cells. 24 h later (d2), second LDH assay was performed using 50 μL supernatant before performing the Cristal Violet assay.

(**B**) <u>Cell toxicity of 16HBE cells after TatRI-iCAL36 incubation.</u> Cells were incubated with TatRI-iCAL36 at the indicated concentrations (1 μM = [1], 10 μM = [10] and 40 μM = [40]) for 3.5 h. Non-transfected cells were used as negative control and Triton-treated cells were used as positive control. Graphical representation of data obtained from duplicates from three independent cell cultures (mean ± SD, n = 3). Cell toxicity is determined at values ≤20%.

<u>(**C**) Cell viability of 16HBE cells after TatRI-iCAL36 incubation.</u> Cells were incubated with TatRI-iCAL36 at the indicated concentrations (1 μM = [1], 10 μM = [10] and 40 μM = [40]) for 3.5 h. Non-transfected cells were used as positive control and Triton-treated cells were used as negative control. Graphical representation of data obtained from duplicates from three independent cell cultures (mean ± SD, n = 3). Cell viability is determined at values ≥80%.
